# Microstructural Parameters for Modelling of Superconducting Foams

**DOI:** 10.3390/ma15062303

**Published:** 2022-03-20

**Authors:** Michael Rudolf Koblischka, Anjela Koblischka-Veneva, Quentin Nouailhetas, Ghazi Hajiri, Kévin Berger, Bruno Douine, Denis Gokhfeld

**Affiliations:** 1 Experimental Physics, Saarland University, P.O. Box 151150, D-66041 Saarbrücken, Germany; a.koblischka@gmail.com (A.K.-V.); quentin.nouailhetas@univ-lorraine.fr (Q.N.); 2 GREEN, Université de Lorraine, F-54000 Nancy, France; ghazi.hajiri@univ-lorraine.fr (G.H.); kevin.berger@univ-lorraine.fr (K.B.); bruno.douine@univ-lorraine.fr (B.D.); 3 Kirensky Institute of Physics, Federal Research Center KSC SB RAS, 660036 Krasnoyarsk, Russia; gokhfeld@yandex.ru

**Keywords:** superconducting foams, YBCO, microstructure, modelling parameters, foam cells, current flow

## Abstract

Superconducting YBa${}_{2}$Cu${}_{3}$O${}_{y}$ (YBCO) foams were prepared using commercial open-cell, polyurethane foams as starting material to form ceramic Y${}_{2}$BaCuO${}_{5}$ foams which are then converted into superconducting YBCO by using the infiltration growth process. For modelling the superconducting and mechanical properties of the foam samples, a Kelvin-type cell may be employed as a first approach as reported in the literature for pure polyurethane foams. The results of a first modelling attempt in this direction are presented concerning an estimation of the possible trapped fields (TFs) and are compared to experimental results at 77 K. This simple modelling revealed already useful information concerning the best suited foam structure to realize large TF values, but it also became obvious that for various other parameters like magnetostriction, mechanical strength, percolative current flow and the details of the TF distribution, a refined model of a superconducting foam sample incorporating the real sample structure must be considered. Thus, a proper description of the specific microstructure of the superconducting YBCO foams is required. To obtain a set of reliable data, YBCO foam samples were investigated using optical microscopy, scanning electron microscopy and electron backscatter diffraction (EBSD). A variety of parameters including the size and shape of the cells and windows, the length and shape of the foam struts or ligaments and the respective intersection angles were determined to better describe the real foam structure. The investigation of the foam microstructures revealed not only the differences to the original polymer foams used as base material, but also provided further insights to the infiltration growth process via the large amount of internal surface in a foam sample.

## 1. Introduction

Superconducting YBa${}_{2}$Cu${}_{3}$O${}_{y}$ (YBCO) open-cell foam samples [1,2,3] are interesting materials for a variety of applications (fault current limiters, trapped field magnets, space applications [4,5,6,7,8,9,10,11]) due to their various, unique properties, which include low sample weight, a very effective oxygenation process, excellent thermal properties allowing for quick cooling, excellent mechanical properties, the possibility for easy shaping of the samples and straightforward upscaling of the sample size. The cooling effectivity, effective oxygenation and sample upscaling have previously been demonstrated in [3,12,13]. Measurements of trapped fields (TFs) in superconducting YBCO foam samples [13,14,15] have revealed a more complicated TF pattern than those of conventional bulk samples. The presence of several small peaks in the TF patterns was ascribed to the compression of local current loops in the sample. Furthermore, it was found that these small peaks do not arise from the same positions in the sample for subsequent TF experiments. These observations demonstrated that current flow through a superconducting foam sample is complicated due to the 3D arrangement of the foam struts, so that local current loops can be formed. Another observation is that the mechanical strength of the foam samples is superior to that of conventional samples. The foams are less prone to cracking caused by magnetostriction [16,17]. The typical internal cracks of bulk samples due to the oxygenation process [18,19] do not exist in the foams.

Thus, to enable the use of superconducting foam samples in various applications, a better understanding of their superconducting and mechanical properties is essential. Computer modelling may be used to assist in reaching this goal. This approach has been utilized in delineating the mechanical and thermal properties of polymer and metal foam materials [20,21,22,23,24,25].

The already existing modelling approaches for polyurethane foams [21,22,23,24,25] may provide a useful starting point. It has been shown that modelling of foam samples with a regular succession of cells and a simple cell geometry (the Kelvin cell) as a first approximation does permit reasonable prediction of properties. However, for a better description of the mechanical properties of polymer and metallic foam materials [26,27,28,29,30,31], it is necessary to improve the model using parameters from the real foam structure. Therefore, it is essential to properly evaluate the microstructure of the superconducting foams to obtain information to improve the modelling. This is particularly important since the double-step fabrication process (polyurethane foam → ceramic Y${}_{2}$BaCuO${}_{5}$ (Y-211)-foam → superconducting YBCO foam) may alter the original foam microstructure. Although to a first approximation, the ceramic material will mimic the structural arrangement of the polymer foam. Furthermore, the infiltration growth (IG) process [32,33] applied to the foam sample will create a unique microstructure, which is not seen in bulk superconductors [34] due to the large amount of internal surfaces in the foam structure.

To achieve a better understanding of the details of the foam microstructure, a thorough analysis of the real foam microstructure of open-cell, superconducting YBCO foams has been performed. Digital optical microscopy, scanning electron microscopy (SEM) and electron backscatter diffraction (EBSD) have been used to identify the structural parameters important for the modelling of superconducting foam samples.

## 2. Experimental Procedures

### 2.1. Sample Preparation

**Figure 1:**
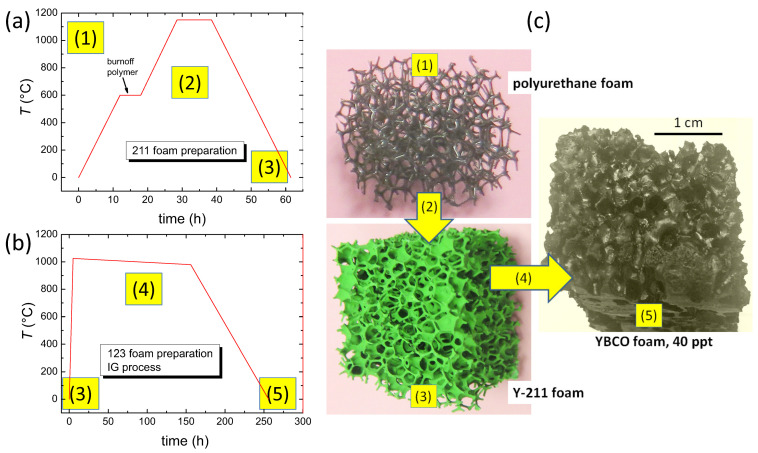
(**a**) Heat treatment to obtain the Y211 foam, (**b**) heat treatment (IG-process) converting the Y-211 foam to an superconducting YBCO foam. (**c**) Illustration of the foam samples at different process stages (1), (3) and (5).

For better understanding the microstructure of the superconducting YBCO foams, the details of the fabrication steps must be considered. The preparation process of the superconducting YBCO foam samples is a two-step process:

(i) Starting from commercially available polyurethane foams (1) [35], which define the porosity and other structural parameters of the final product, the sample is covered with a slurry of Y-211 powder dissolved in polyvinyalcohol (PVA) and water. To form ceramic Y-211 foams, a heat treatment is required to burn off the polyurethane and to compact the Y-211 ceramic (2). This is illustrated in the temperature program shown in Figure 1a.
(ii) The green Y-211 foam (3) is then converted into the YBCO superconductor using the IG process [32,33] using a Nd-123 seed crystal on top and a liquid source consisting of a 1:1 mixture of Ba and Cu oxides with an overall stoichiometry of Ba${}_{3}$Cu${}_{5}$O${}_{y}$ and additional 123 powder placed beneath the Y-211 foam. When being heated above the eutectic temperature (1010 ${}^{\circ }$C, (4)), the liquid phase infiltrates the Y-211 foam by capillary action [36]. The seed crystal ensures an overall texture of the superconducting foam sample like in the case of bulk superconductors. Finally, the Y-211 foam is fully converted to the 123-phase (5) in a slow-cooling process.

Thus, the superconducting YBCO foams owe their specific properties to the IG process applied. The liquid phase, which moves along the foam struts from bottom to the top of the sample, plays an essential role for the appearance and shape of the foam struts. This will be investigated in detail using electron microscopy and digital optical microscopy in the following sections. The samples investigated in this study were prepared at RWTH Aachen, Germany, as well as new ones, which were fabricated using the same process parameters.

### 2.2. Microscopy Investigations

Digital optical microscopy was performed using a Keyence VHX-5000 microscope [37] with a large depth of field and long observation distance (most images were taken with 100× magnification), enabling 3D imaging and digital image processing/analysis. The images were treated by the built-in analysis software for calibration and size measurements. Using the possibility to record the image processing steps, an automated routine for determining, e.g., the window sizes, can be generated. This enables a large number of images to be processed in reasonable time. Additional image analysis was performed using ImageJ [38] and WSxM software [39].

SEM images were taken using a field emission scanning electron microscope (JEOL 7600F) and a JEOL 7000F SEM microscope operating at 20 kV with a working distance of 10 mm. EDX analysis was performed using an EDAX ZAF system with a SUTW sapphire detector.

The EBSD orientation imaging was performed in a JEOL 7000F SEM microscope equipped with a TSL (TexSEM Labs, UT [40]) analysis unit. The Kikuchi patterns were generated in reflection mode [41] at 15 kV, and were recorded by means of a DigiView camera system. To perform crystallographic orientation mapping, the electron beam was scanned over a selected surface area and the resulting Kikuchi patterns were indexed and analyzed automatically. Automated EBSD scans were performed with an EBSD step size down to 50 nm.

The sample surfaces for EBSD analysis were mechanically polished using SiO${}_{2}$ grinding papers, followed by mechanical polishing with diamond pastes (3 μm down to 1/4 μm diamonds) using ethanol as lubricant. The final step consisted of polishing the surface using colloidal silica with 40 nm particles (Struers OP-S solution). More details on the sample surface preparation steps can be found in [42]. An additional low-angle (5${}^{\circ }$) Ar ion-polishing step (5 keV, 5 min) could be applied to the sample surface after the mechanical treatment. This process increases the image quality (IQ) of the resulting Kikuchi patterns and removes mainly adhered particles on the sample surface.

## 3. Results and Discussion

### 3.1. Scanning Electron Microscopy

**Figure 2:**
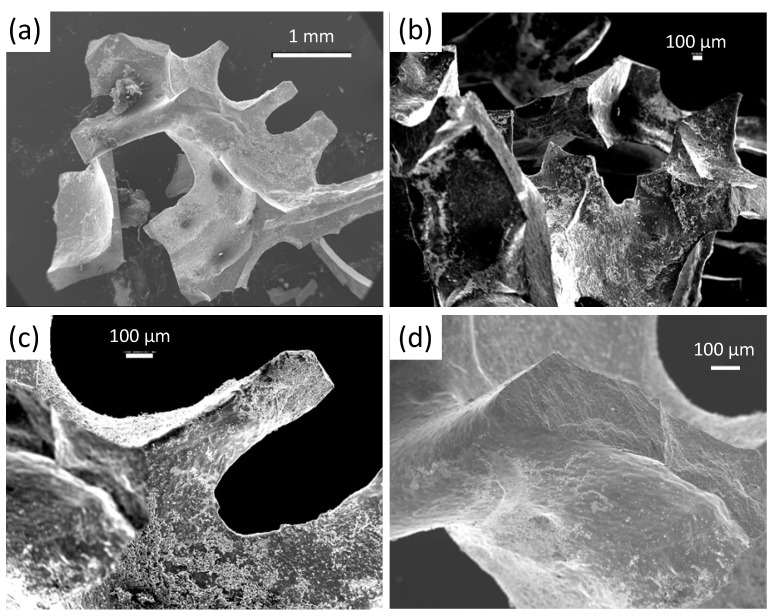
(**a**–**d**) SEM images at low magnification of broken-out foam strut pieces from a superconducting YBCO foam (magnification 25× (**a**), 35× (**b**), 85× (**c**) and 95× (**d**)), giving information of the real foam microstructure and the various internal surfaces existing in the open-cell foam structure. Note the rough character of the as-grown strut surfaces of the YBCO foam. The images further demonstrate the irregular shape and size distribution of the foam struts, which need to be modelled.

**Figure 3:**
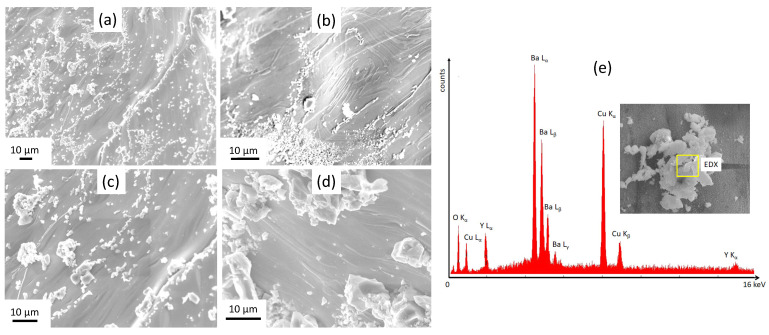
(**a**–**d**) SEM images (various magnifications ranging between 650× and 2200×) showing details of the internal surfaces of the foam struts and vertices. The particles seen on the strut surfaces are Ba${}_{3}$Cu${}_{5}$O${}_{y}$ particles as determined by EDX analysis (**e**). These structures and particles are caused by the capillary flow of the liquid phase during IG-processing, which is not seen in conventional bulk samples.

Figure 2a–d present SEM images in various magnifications of foam strut pieces broken-out from a bulk YBCO foam. These pieces reveal directly the complicated and irregular arrangement of the foam struts and their vertices within the foam sample (a,b), exhibiting various ligament (strut) lengths, ligament cross sections and intersection angles. In the following, a foam strut denotes an entire broken-out section and the ligament is the material section between two adjacent nodes. In case of higher magnification (c,d), the images further reveal a certain characteristic structure of the foam strut surface. This structure was caused by the capillary flow of the liquid phase along the Y-211 struts, which left a rough and rugged surface, comprising several particles, particle clusters and flow structures. In Figure 3a–c, more details of the strut surface structure are presented using higher magnification, illustrating the distribution of the Ba${}_{3}$Cu${}_{5}$O${}_{y}$ particles. Here, it must be noted that the foam sample offers a high amount of internal surfaces, which do not exist in conventional bulk samples prepared using the same IG-processing method. Figure 3a–c reveal that a large number of Ba${}_{3}$Cu${}_{5}$O${}_{y}$ particles with typical size ranging between 0.5 and 3 μm are distributed in an irregular fashion on the strut surfaces as individual particles or as particle clusters. EDX analysis (d) revealed that the particles are mainly Ba${}_{3}$Cu${}_{5}$O${}_{y}$, that is, particles of the pure liquid phase used in the IG-processing. Besides these particles, the SEM images indicate distinct growth steps and some characteristic patterns on the strut surfaces, which are caused by the capillary flow of the liquid phase. These patterns were shown by EDX to possess a higher content of Y as the particles on the surface. All this information gained on the strut surfaces gives valuable input to better understand the details of the IG-process, which are not seen in conventional bulk samples. A closer investigation of these particles on the strut surfaces and their possible effect on the flux pinning properties will be presented in a forthcoming study [43].

**Figure 4:**
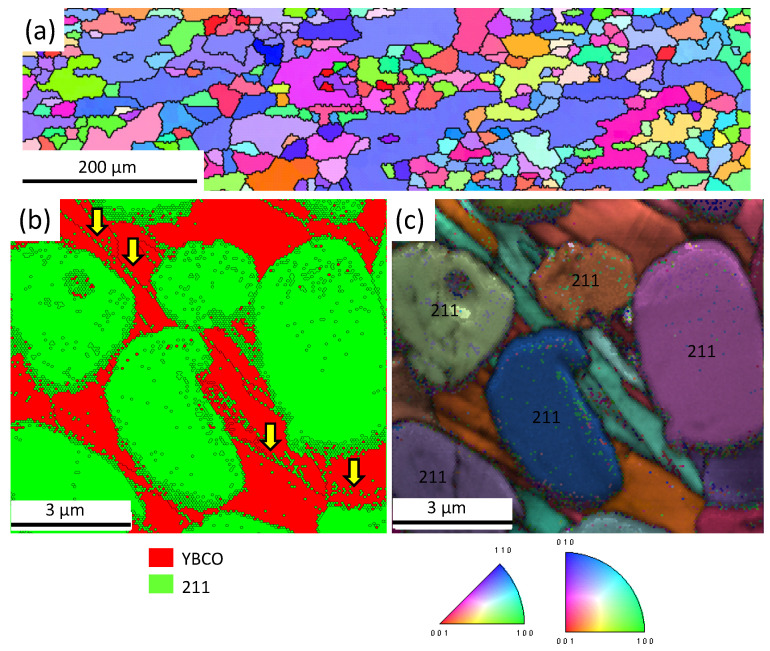
(**a**–**c**) EBSD orientation imaging of foam strut pieces with mechanically polished surfaces. The scan areas are selected to be entirely inside the struts. (**a**) Overview measurement (scan area ∼ 1 mm × 200 μm) along a foam strut ligament. Orientations are given perpendicular to the sample surface, i.e., in [001]-direction. The EBSD stepsize for this measurement was 2 μm. Both phases (YBCO and Y-211) are plotted together. The color code for the orientation mapping is given below image (**c**). (**b**) Phase mapping (red—YBCO, green—Y-211) of a 10 × 10 μm${}^{2}$ scan area. Several large and many tiny Y-211 particles are embedded within the YBCO matrix. The EBSD-detected GBs are indicated using thin, black lines. The yellow arrows point to the tiny Y-211 particles found in groove-like structures in the foam strut. (**c**) Inverse pole figure (IPF) map with overlaid image quality (IQ) mapping, recorded with high magnification and an EBSD stepsize of 50 nm. Note here that the YBCO matrix shows orientations up to 60${}^{\circ }$ off the [001]-orientation which reflects the 3D-orientation of the strut within the bulk foam. The Y-211 particles reveal several different orientations.

Figure 4a–c present EBSD measurements on polished foam strut surfaces. Figure 4a is an overview measurement of the orientation distribution (inverse pole figure, abbreviated IPF) in [001]-direction along a foam strut ligament. The scan area is selected to be fully *inside* the foam strut. Furthermore, as the strut surface has underwent mechanical polishing, the particles on the surface are removed, so only two phases (YBCO and Y-211) are present. The crystallographic orientations are given perpendicular to the sample surface, i.e., in [001]-direction. Note that there is practically no orientation in [001]-direction (red) like in the case of a bulk superconductor pellet, which is due to the original 3D orientation of the strut *within* the bulk foam. Thus, the present observation is *not* contradicting the overall texture of the bulk foam as verified by neutron diffraction in [44,45]. The YBCO matrix consists of elongated grains showing various orientations 30–60${}^{\circ }$ off the [001]-orientation. In contrast, the large Y-211 particles seem to be randomly oriented. In Figure 4b,c, EBSD-mappings with high resolution are shown using an EBSD stepsize of 50 nm. Figure 4b presents a phase map with YBCO plotted in red and Y-211 in green. Furthermore, the EBSD-detected grain boundaries (GBs) are marked by thin black lines. There are several large Y-211 particles showing some substructures. Many small grains are located along the edges of the big Y-211 grains, but also very tiny Y-211 particles covering just one EBSD step [46] can be detected. This is an indication of a small particle size ∼50 nm. Large numbers of such tiny Y-211 particles are found to fill the GBs between the YBCO grains (yellow arrows point to several such locations). This type of arrangement of Y-211 particles was not observed in any bulk YBCO pellets as already discussed in [47]. The inverse pole figure (IPF) orientation map, overlaid with the image quality (IQ) information, presented in Figure 4c reveals that only two main orientations exist for the YBCO grains within the scanned area. In contrast, the large Y-211 grains are practically randomly oriented.

All observations of grain orientations within the foam struts are important to understand the flow of superconducting currents in the superconducting foam samples. Most importantly, the EBSD data reveal that the currents within a foam strut must cross several GBs, which poses a severe limitation as seen in the time-dependent TF measurements performed in [48], even though the overall foam sample has texture introduced by the seed crystal, which was verified by neutron diffraction experiments [44,45]. The possible influence of the Ba${}_{3}$Cu${}_{5}$O${}_{y}$ particles located on the strut surfaces on the flux pinning properties was not yet studied, but it is obvious that the capillary transport of the liquid phase along the foam struts has altered the shape and appearance of the final YBCO foam struts as compared to those of the polyurethane foams, from which the processing had started.

### 3.2. Digital Optical Microscopy

**Figure 5:**
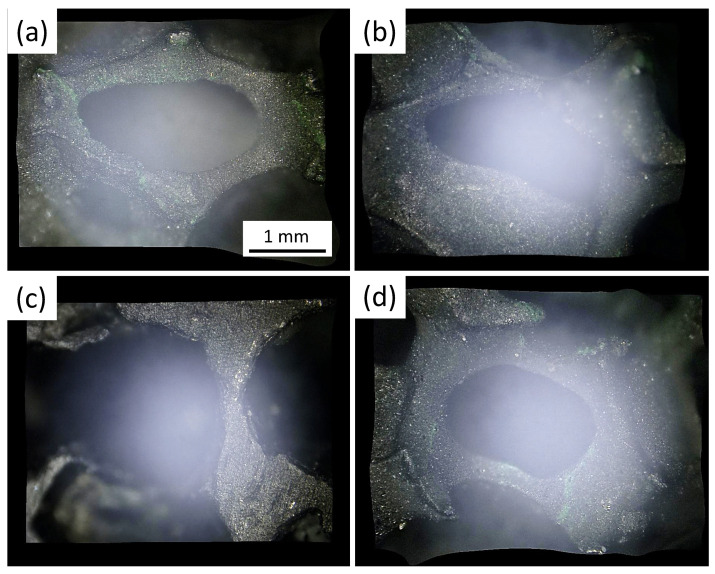
(**a**–**d**) Optical raw images of foam struts, ligaments and windows in various positions within the bulk foam sample taken by a Keyence VHX-5000 microscope, allowing a large focal depth. These images provide an impression of the local variation of the foam windows, struts and ligaments, which need to be modelled.

**Figure 6:**
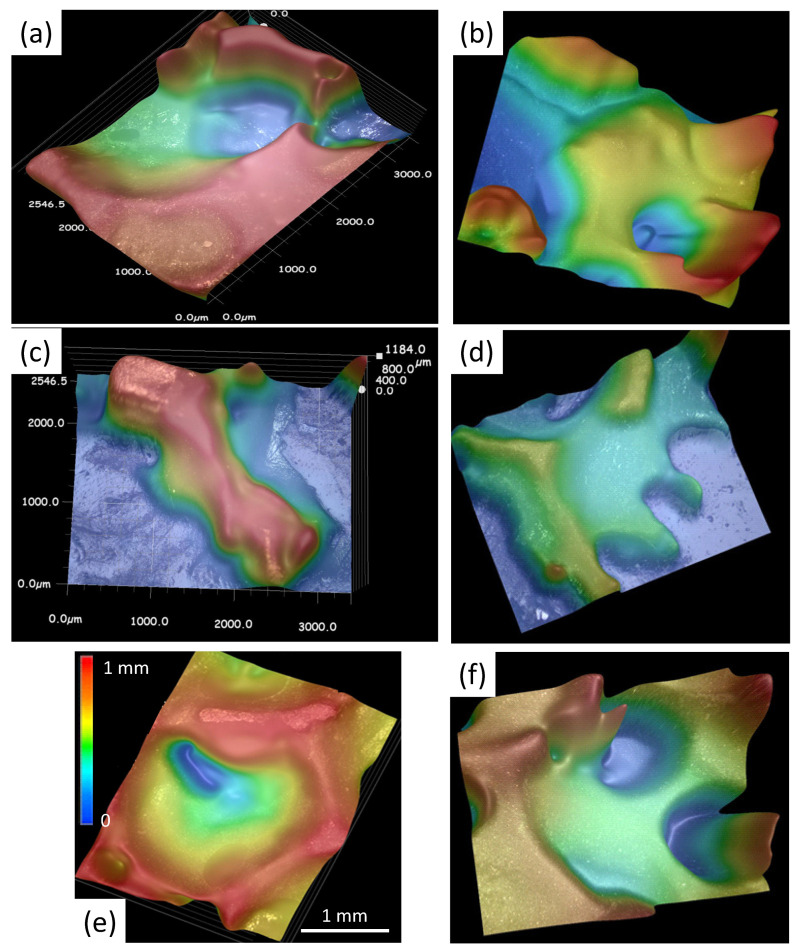
(**a**–**f**) Digitally processed images of foam windows, struts and ligaments in various positions within the bulk foam sample. Images (**a**,**c**) show the calibrated size data, and (**e**) gives the extracted values for *x* and the color code for *z*. These images are the base for the analysis presented in Figure 7 below.

Using digital optical microscopy, a large number of images were taken to study the various shapes of the cells building up the YBCO foam sample. Using the specific focal depth variation enabled to obtain processed 3D images of the foam cells. All the images presented here were collected on full-size foam samples by means of a Keyence VHX-5000 microscope. The included image analysis software enabled a semi-automated analysis. Overall, about 200 such images were collected and analyzed. Figure 5a–d present untreated raw images of several foam cells with a high focal depth, showing typical foam struts and windows in their original location within the foam sample. These images serve as the input for the following analysis. The image-processed images of the foam microstructure as shown in Figure 6a–f give an impression of the real arrangement of the foam struts, windows and cells. This analysis provides the necessary input data (ligament length, intersection angles and window size) to the statistical analysis to obtain proper parameters for the modelling.

### 3.3. Discussion of Modelling Approaches and Parameters

The first issue is a discussion about the sample density and the amount of superconducting material in a foam sample. As the superconducting foams are prepared via the IG-process, the foam struts share their properties with the IG-processed bulks, even though there are certain differences in the microstructure. In [10], the densities of various melt-processed samples was discussed leading to different levitation forces. The density of the melt-textured samples ranged between 5.88 and 6.17 g/cm${}^{3}$, the tabulated theoretical density of YBCO being 6.4 g/cm${}^{3}$. The difference is due to the presences of pores and cracks, and also the lower density of Y-211 particles which make up to 40% of the material. In the case of foams, the presence of pores reduces the density to 1/5⋯1/10 of the density of the bulks, depending on the porosity of the original polyurethane foam (20⋯40 ppi).

A second issue concerns the underlying polyurethane foams, as their internal structure provides the base for the superconducting foams. Up to now, only products from one company were used for the superconducting foam preparation [35], so the present analysis focuses on this type of open-cell, reticulated polyurethane foam. The manufacturer gives the pore size in ppi (pores per inch) with a typical variation of ±5 pores. However, when comparing the foams of different makers, the geometry of the struts, windows and cells may be considerably different, which may lead to different mechanical behavior of the final samples. This was pointed out recently in Refs. [49,50], where additive manufacturing was employed to create artificial foam structures, and in Ref. [51], where porous sintered tin-bronze alloys were studied. Different microstructures of the foams may match specific application requirements (e.g., filters, heat exchangers, etc.). Such different microstructures were also observed in our case when selecting possible other suppliers of polyurethane foam materials, so we sticked to the products of the original maker to limit the variations.

The previous modelling of polyurethane foams clearly revealed that it is important to properly model the real foam microstructure in order to achieve reasonable results for the mechanical properties of such foams as the real foam samples showed higher mechanical strength. Thus, we have analyzed about 200 such images to obtain a reasonable parameter set characterizing the YBCO foam samples.

**Figure 7:**
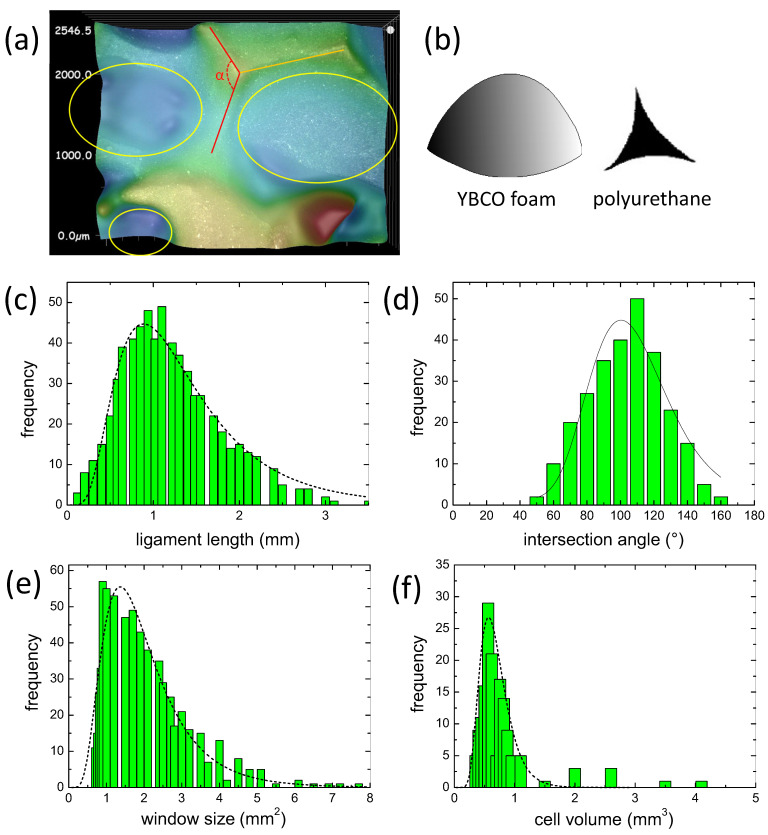
(**a**) Definitions of the ligament length (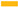), the intersection angle α (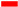) and the window size (yellow ellipses). (**b**) presents the typical cross section found by profile analysis for the YBCO foam (left) compared to a sketch of the cross section following Jang et al. [22]. (**c**–**f**) Statistical distribution analysis of the ligament (strut) lengths, the window size, the intersection angles and the cell size. The black dashed lines indicate log-normal fits to the data, and a normal fit in (**d**).

Figure 7a presents the definition of the ligament length (orange line), the intersection angle α (red lines) and the foam windows (yellow ellipses). Ligament lengths and intersection angles were determined manually, whereas the window size was determined via the Keyence software with selected border values.

The Kelvin cell geometry has already been used by many researchers to represent foam structures [20,21,22,23,24,25]. This geometry consists of six square and eight hexagonal faces and is capable of partitioning the space into identical equal-volume units with minimal surface energy. However, in this model all foam struts are identical, and the nodes, where the struts interconnect, are quite simplified. Although such Kelvin cell models have proven to be efficient and useful to model the mechanical response of cellular materials in the literature, the geometry of the Kelvin cell does not comply with a real foam topology. The cells of real foams are irregular polyhedra with anywhere from 9 to 17 faces when regarding nearly monodisperse foams. The material is concentrated in the nearly straight ligaments and in the nodes where they intersect. Therefore, the mechanical properties of foams depend strongly on the microstructure realized, and thus, on the basic properties of the base material used to prepare the foam sample.

The specific part of the microstructure, which is relevant for the mechanical properties of the foam, is the shape and geometry of the various nodes [22] as there is a large amount of material concentrated. Jang et al. further showed that the cross section of a ligament changes along its length, being relatively small in the center and growing towards the ends, which is due to an elongation when forming the polyurethane foam. For their polyurethane foam sample, they found a characteristic three-cusp hypocycloid cross section of Plateau borders, which is illustrated in Figure 7b(right). From our present analysis, it is obvious that the flow of the liquid phase in the IG-process has altered the cross section of the foam struts or ligaments. The shape, as revealed by a large number of profiles taken from the optical images, has changed to a paraboloid type (see Figure 7b(left), and the sharp cusps of the original polyurethane foam have almost vanished. We further note that there is a spatial variation of the strut shape within the foam sample. From the bottom side, which was closest to the liquid source, the shape is found to change from the parabolic type (which contains more material on the ligands) to a more cusp-like type at the top face of the foam sample, which indicates the amount of liquid which was present during the processing. Previous work on flux pinning and critical currents in the foam sample [52,53] also revealed a clear dependence on the position within the original foam, and the TF measurements on all sides of a foam sample demonstrated that the top side was the weakest one [13]. Thus, this finding is another important issue for improving the properties of future YBCO foam samples.

Figure 7c–f present the results of our image analysis. Figure 7c gives the histogram of the ligament lengths from 601 ligaments, ranging between 0.12 and 3.5 mm. All data were fitted by log-normal functions with four parameters:

(1) $$y=y_{0}+\frac{A}{\sqrt{2\pi }wx}\cdot e^{-{\left(\frac{\ln(x/x_{c})}{\sqrt{2}w}\right)}^{2}}\;\;\;\;,$$

as indicated in the graphs by dashed black lines with $y_{0}=$ 0. The resulting parameters are $x_{c}=$1.2 mm, w= 0.55 and A= 53. Similar to the data presented by Montmimy et al. [21], the data fall in a right-skewed distribution, which is common for natural systems. In Figure 7d, the intersection angle histogram is presented, which represents a nearly normal distribution. The mean angle determined here is 104.1${}^{\circ }$ (standard deviation 1.25${}^{\circ }$), which is smaller than the angle found by Montmimy et al. in their polyurethane foams, and clearly smaller than the tetrahedral angle of 109.5${}^{\circ }$. The foam window size distribution is shown in Figure 7e, yielding $x_{c}=$ 1.25 mm, w= 0.26 and A= 35. Like in the case of Montmimy et al., this distribution is again right-skewed, but much stronger than the distribution of the ligament length, which says that there is a bigger variation of the window sizes as compared to the ligament lengths. Finally, Figure 7f gives the cell size histogram (155 cells analyzed) with the parameters $x_{c}=$ 0.84 mm, w= 0.12 and A= 8. Altogether, this analysis demonstrates that the real foam structure is clearly different from a simple Kelvin cell, and the parameter set obtained will be useful for generating a true foam model. The important finding here is the fact that the cross sections of the YBCO foam are distinctly different from the polyurethane foam, and also from the intermediate Y-211 foam.

### 3.4. First Modelling of Field Cooling and Trapping

**Figure 8:**
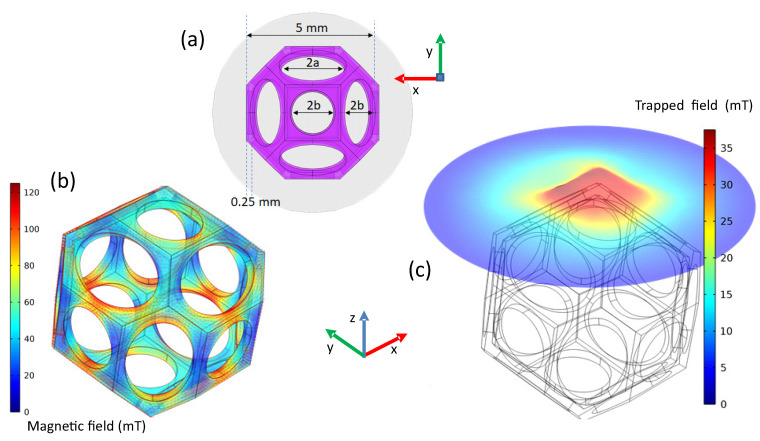
First modelling of trapped fields of a foam using the Kelvin cell, the dimensions of which are given in (**a**). (**b**) presents the distribution of the local magnetic field after field cooling in a field of 50 mT, and (**c**) gives the trapped field distribution at 2.75 mm above the foam.

Finally, a first attempt to model the properties of a superconducting foam sample is presented here. The modelling was started using a Kelvin type model of a foam cell as depicted in Figure 8a, which is the most simple representation of a foam cell. The selected parameters are as follows: foam cell 5 mm wide, 0.25 mm thick struts, and the cell consists of circles with radius b= 0.833 mm and ellipses with major axis a= 1.25 mm and minor axis b= 0.833 mm. The foam is arbitrary tilted by 10${}^{\circ }$ from the *z*-axis and 10${}^{\circ }$ along the *x*-axis. A surface representing the field mapping area is placed at 2.75 mm above the foam. The calculations were performed using classical **H** formulation coupled with circuit coupling or external field [54], or alternatively, using the **A–H** coupled formulation [55] implemented in the PDE module and the global equation module in COMSOL Multiphysics version 5.2a. The electrical parameters to simulate the YBCO foam sample use the power law $E\left(J\right)=E_{c}{\left(J/J_{c}\right)}^{n}$ with the parameters $E_{c}=$ 1 μV/cm, n= 20, and a critical current density $J_{c}=$ 1000 A/mm${}^{2}$, corresponding to the measurements on foam struts of Ref. [53]. Field cooling in a field of $B_{\mathrm{app}}=$ 50 mT was modelled after reducing the field linearly towards 0 within 10 s, and the end of the process is depicted in Figure 8b. Finally, a simulated trapped field distribution is presented in Figure 8c, which gives a nearly homogeneous TF distribution.

The results of this first TF modelling using a simple Kelvin cell already gives reasonable output as compared to the measured TF values presented in [13]. An interesting finding is the reddish areas along the foam struts as seen in Figure 8b, depicting the field-cooled flux distribution. This observation implies that the thinnest sections of the foam ligaments form bottlenecks for the superconducting currents in the foam, experiencing the highest magnetic fields. Thus, the present shape of the foam ligaments is by no means well-suited to sustain a high critical current density. Therefore, these results point out that a more regular shape of the foam cells and, correspondingly, regular orientation of the foam ligaments would bring a better current distribution (as the main current flow takes place within the Cu-O-planes) in a superconducting foam sample. Such kinds of structures could be produced by 3D printing/additive manufacturing as was already demonstrated in Refs. [56,57,58], which could significantly improve the TF performance of porous superconducting materials [59,60,61]. So, the first modelling of the trapped fields in superconducting foam samples using the Kelvin cell-type approach already delivered an important result for future applications of such foam samples. This is a first hint that a simple approach to model the superconducting performance of a foam sample may be sufficient for some specific purposes. Introducing irregular cell sizes and a variation of the ligament thickness using the data elaborated here will not lead to higher calculated TF values, but may better reproduce the effects of current loop compression, the percolative current flow, etc. Thus, the modelling of the TF fields represents only one facette of the various properties being important for applications, which must be well designed in the view of possible applications. One example for this is energizing the superconducting foam sample with pulsed currents in a separate coil. This will cause magnetostriction effects, resulting in strong forces on the foam structure. To model this situation and also the mechanical properties when applying the foam in a flux-pinning docking interface (FPDI) system as described in [11], a further developed model making use of the cell parameters determined here, will undoubtedly be necessary to find an optimized foam structure (porosity, amount of superconducting material, cell and ligament diemsions) for a given type of application. It will be very interesting to see which sample property requires a simple or an extended modelling approach. For such extended modelling, a well-suited direction to go for may be the lattice geometry approach as described in Ref. [62]. This will be investigated in subsequent works.

Regarding the very specific microstructure of the superconducting YBCO foams, one must note here that the foam struts of the superconducting foams have a different shape as compared to the starting polyurethane foams. Strongly linked to the capillary flow of the liquid phase during IG-processing, a specific microstructure of the superconducting foams results. Tiny Y-211 particles are located mostly in groove-like channels within the YBCO matrix [34], and the strut surface exhibits a presence of Ba${}_{3}$Cu${}_{5}$O${}_{8}$-particles stemming form the liquid source, which may also contribute to the flux pinning. Such additional particles on the sample surface were not seen previously in the commonly prepared bulk samples. All these details are essential for the superconducting performance of the foam samples. Thus, the final model of a superconducting foam must consider all these specific details of the foam microstructure.

## 4. Conclusions

A detailed analysis of the microstructure of superconducting YBCO foams revealed several interesting details, including the effects of the capillary flow of the liquid phase in the IG-process along the foam struts, the formation of Ba${}_{3}$Cu${}_{5}$O${}_{y}$ particles and flow patterns on the internal surfaces of the foam and the formation of tiny Y-211 nanoparticles located in channel-like structures within the YBCO matrix. SEM and digital optical microscopy provide valuable input to better understand the real foam structure, also revealing clear differences to the polyurethane foams, which served as the base material. The first modelling of the magnetic properties was performed using the regular Kelvin cell approach and the **H**-formulation, which could already reproduce the basics of the trapped field measurements. Furthermore, the model data suggest that a uniform distribution of cell sizes could provide better TF performance as the irregular structure of the present YBCO foam material.

The material of this manuscript was presented at the HTS modelling 2020 workshop, Nancy, held online in 22–23 June 2021. This work is part of the SUPERFOAM international project funded by ANR and DFG under the references ANR-17-CE05-0030 and DFG-ANR Ko2323-10, respectively.

## Data Availability

Datasets obtained and analyzed during the study are available from the corresponding authors on reasonable request.
